# Hospital Meetings, &c.

**Published:** 1900-03-31

**Authors:** 


### HOSPITAL MEETINGS, &C.

### HOSPITAL AND HOME FOR INCURABLE CHILDREN.

Canon Duckworth presided, as usual, over the annual meeting of the friends and governors of the Hospital and Home for Incurable Children, 2, Maida Vale, which was held at the hospital. The report was read by the hon. secretary, and the financial statement by the hon. treasurer; both were seconded by Mr. Fisher, and carried unanimously. Neither showed much deviation from previous year. The Committee of Management having accepted some of the little patients at reduced fees the receipts under that heading were less than before. The list of subscribers is still too small to guarantee the maintenance fund, and consequently a portion of the legacies received had had to be used instead of invested. The ordinary income for the year was £852, the extraordinary £1,050; the expenditure was £1,261, and £1,049 was invested. It has unfortunately been found necessary to underpin the remaining half of the foundations of the house, which will necessitate considerable outlay. The reports were accepted and approved. The election of Mr. F. J. Lewis and Mr. Saffery as members of the committee was confirmed, and Mr. T. Aylmer Lloyd has resigned his seat. The vote of thanks to the honorary officers was moved by General Lowrie in appreciative terms. Mr. Bonnor-Maurice has resigned the position of honorary treasurer, and Mr. Francis J. Saffery has accepted it. The laws for the government of the institution have been carefully revised, obsolete regulations have been removed, and the remainder brought up to date. The alterations were chiefly clerical, and as copies of the new laws had already been circulated amongst the Governors, the Chairman put them unread for adoption by the meeting, and they were carried unanimously. The proceedings concluded with the singing of the National Anthem.

### ST. MARY'S HOSPITAL.

Colonel Bird presided at the annual meeting of St. Mary's Hospital, Paddington, on Friday last, 16th inst. The proceedings were of the customary brief and businesslike character, the resolutions being adopted without discussion or comment. From the accounts presented it appeared that the ordinary income for the past year was £11,766, and the ordinary expenditure £23,121. The extraordinary income was £33,513, but of this £3,500 consisted of endowment donations and £5,100 of legacies directed by the testators to be invested. The extraordinary expenditure was £6,209, of which £5,514 was on building improvements, and £501 on repairs. The report stated that the year had been an eventful one, marked by the carrying out of important internal improvements. In the last report it was stated that steps were contemplated for replacing the £19,650 advanced from the general maintenance fund capital for the completion of the new out-patients' department forming the basement of the Clarence Memorial wing. A bazaar was organised on a large scale, with a ladies' and a gentlemen's committee for furnishing stalls and for collecting donations. The effort resulted in the raising of £22,997, a larger sum than had ever before been obtained for the hospital. The bazaar was held at the Hotel Great Central, the whole ground floor being placed at the committee's disposal. An anonymous friend defrayed the whole of the expenses, while Messrs. Maple and the proprietors of the hotel supplied, free of charge, the decorations, fitting up of the stalls, and the refreshments. The estimated cost of completing and furnishing the Clarence wing is £50,000, and towards this a reserve fund had been started with £2,947, balance of proceeds of the bazaar after repayment of the loan from the Maintenance Fund. In spite of the claims of the war, it was hoped that the completion of the new wing would not long be delayed; much difficulty was constantly being experienced in finding room for urgent cases, while two houses had had to be taken in the neighbourhoood to provide accommodation for the nursing staff. During the year the medical wards and casualty rooms were refloored with teak. The reconstruction and refitting of the operating theatre had been effected, and the kitchen and offices greatly improved and enlarged by structural alterations and supplied with a complete set of apparatus. The number of out-patients treated was again larger than ever, despite the maintenance of the inquiry system instituted in 1897. The proportion of cases found to be unsuitable was reduced to 1·41 per cent., little more than half that of the previous year. One sister and eight nurses had been added to the staff during the year, while three sisters had left; one only temporarily having obtained special leave to take duty on the Army Nursing Reserve in South Africa. Three of their surgeons were also with the field force, in addition to a number of former students. After 15 years of service, Mr. G. P. Field had resigned his office of dean of the Medical School, and had been succeeded by Dr. H. A. Caley. Mr. Field's enthusiasm and unremitting labour were warmly recognised. The usual compliment was paid to the chairman on the termination of the proceedings.

### THE NEW ITALIAN HOSPITAL, QUEEN SQUARE.

The Italian Hospital in Queen Square, of which the new buildings were opened last week by the Italian Ambassador, was founded in 1884 by the late Commendatore Ortelli, who, as the pressure on the hospital's resources grew from year to year, continued to support it most generously, providing for enlargements, and before his death making full provision for the cost of the new building. Mrs. Ortelli, who worked hand-in-hand with her husband, has since his death devoted herself to carrying out his wishes on its behalf, and it was a graceful recognition of the benefits thus rendered to the Italian community in London that was made by King Humbert, in the unexpected presentation of a gold medallion from his Majesty to Mrs. Ortelli by the Ambassador at the opening ceremony. The hospital, though primarily intended for "Italian speaking peoples," is open, so far as its capacities allow, to the sick and suffering of every nationality; as the Ambassador happily said, "On the façade of this building can be inscribed 'Charity knows no restriction of country.'" It contains accommodation for some forty beds, and for a large number of out-patients. Built at the corner of Queen Square and Devonshire Street, the new building has an imposing exterior of red brick, with white stone mullions, pilasters, &c., and inside is bright and airy. The basement is occupied with offices and kitchens; the first floor contains the out-patient department, board-room, house surgeon's rooms, dispensary, secretary's room, &c., and also the operating room and a small surgical ward for two beds. The first and second floors are devoted respectively to men's and women's wards, one large ward on each floor, with several single-bedded wards for special cases and others for children, with an isolation cut off from the building by an open passage

The bath rooms and lavatories are carefully detached from the wards in a special turret. The floors are of concrete or terrazzo in the corridors, the wards being floored with oak, and all the corners are rounded. On the third floor are the sisters' dormitories, and dining, sitting, and sewing rooms, with a small infirmary for their special use, and a circular and domed chapel. A covered way leads to the flat roof, which will make a pleasant retreat in fine weather for convalescents, being provided with sheltered seats. From her private purse Mrs. Ortelli has defrayed the cost of electric lighting, a passenger lift, and other additions to the comfort and convenience of the hospital. Only one ward is as yet furnished, but this had an eminently bright and pleasant aspect on the opening day; the hospital will probably be ready for occupation in a few weeks' time, when it will be interesting to see it in working order. The nursing is under the direction of the sisters of St. Vincent de Paul.

### HAMPSTEAD HOSPITAL.

The eighteenth annual meeting of governors and subscribers to the Hampstead Home Hospital and Nursing Institute was held on Saturday, 17th inst., Mr. W. F. Malcolm (the hon. treasurer) presiding. The accounts presented showed ordinary income £2,189 and ordinary expenditure £3,116. Extraordinary expenditure (repairs) was £53, the excess of expenditure over income for the year being £980. During the year the in-patients numbered 356, among which 76 were accident cases. The average number of beds daily occupied was 21, and the average stay 21·5 days. The out-patients, including casualty and dental cases, numbered 2,291, an increase of 73 on the previous year's total. The increase of in-patients over 1898 was 29. Investigation is made to ensure that none but suitable persons are benefited. The Golder's Hill fête on June 1st was a brilliant success. The amount placed to the building fund was £630, in addition to £1,514 given in purses to the Princess Christian. The Bal Poudré on December 8th was another successful achievement of the Ladies' Association, and yielded £157 to the building fund. The appointment of a house surgeon was mentioned as having again received the careful attention of the council. A special grant of £100 from the Prince of Wales's Fund was made on the understanding that steps would be taken to appoint one. Though a substantial and welcome help this would not suffice to defray more than half the expense the step would entail under present circumstances, no suitable accommodation being available in the hospital, and a special appeal was made to Hampstead people to come to the assistance of their hospital. It was announced that the Earl of Mansfield would preside at the biennial festival to be held at the Grand Hotel on May 16th. The Chairman urged the need for a hospital in that locality, since if a slight accident case had to be taken miles to one of the central hospitals it was likely to become a serious one; while if a serious case had to be taken that distance it was very likely to prove fatal. They had not yet secured a site for the new building, but the committee had several in view, and had great hopes that negotiations now proceeding would result favourably. The great central hospitals were established when London was much smaller, and the distances, both for patients and their friends, were now formidable; in addition they had the enormous benefit of the far purer air. Votes of thanks to the medical staff and the Ladies' Association having been cordially voted, and the council and officer re-elected, the proceedings terminated with the customary compliment to the chairman.

### UNIVERSITY COLLEGE HOSPITAL.

The annual meeting of the governors and subscribers of the North London or University College Hospital was held on Wednesday, 21st inst., Lord Monkswell, the treasurer, presiding. The Chairman, in moving the adoption of the report for 1899, pointed out that the committee hoped very shortly to have in occupation the new north-west wing and central block of the new hospital, the latter providing the main staircase, hydraulic lifts, two general and one special operating theatres, and executive offices. The nurses' wing was rapidly approaching completion, and when the north-west wing and central block were completed the south wing of the present hospital would be demolished to make room for the building of the south-east wing. They hoped that the rebuilding would be completed in about two years' time. The Hospital Committee had decided that in future the nursing of patients should be carried on by a matron, sisters, nurses, and probationers, under the committee's own control and under the immediate supervision of a nursing committee, and Miss H. E. G. Hamilton had been selected for the office of matron. These changes in management had rendered it necessary to close the buildings in August last, with the exception of the outdoor maternity department. The new arrangements had proved thoroughly efficient. There had been, he was sorry to say, a slight falling off in the subscriptions for the past year, the amount of £2,375 comparing with £2,487 in 1898. The hospital Sunday Fund had awarded them £1,406, and the Saturday Fund had contributed £312 from its 1898 collection. The committee had also received from the People's Contribution Fund Committee a donation of £500. Various legacies had been received, in which matter the hospital had this year been rather fortunate. The committee, as they would note, recorded with much regret the death of Captain Sir Douglas Galton, for 23 years an active and valued member of the hospital committee; and they had had the gratification of appointing the Marquis of Northampton and Sir John Sterling Maxwell, M.P., to be vice-presidents of the institution. They were glad to find that the appointment of an inquiry officer had resulted in showing that the abuse of the charity was now very small, the number of unsuitable persons applying for treatment being only 2½ per 1,000. The Christmas appeal had resulted in obtaining £47 in new annual subscriptions and £970 in donations; whilst the amount owing to tradesmen on December 31st last totalled £6,599, as compared with £8,029 on the year 1898. In conclusion, the Chairman pointed out that although the only income the committee can rely on at present is £7,500, they have to look forward to greatly increased expenditure. They feared that the charities in connexion with the war would draw off some of the support they had been accustomed to, but they had need for largely increased support owing to the fact that the new and larger hospital now being built would require, when fully occupied, an annual income amounting to £28,000 to £30,000. Their financial state would be more satisfactory if the Permanent Endowment Fund were very largely increased. The cost for the full endowment of a bed or cot is £2,000, but the committee would gladly receive the amount by instalments. The report was then adopted. The total ordinary income for 1899 amounted to £12,067; and the total extraordinary income to £13,793. The total ordinary expenditure was £18,784, and extraordinary expenses, £7,422. A cordial vote of thanks to Lord Monkswell for his interest in the hospital, and for presiding, was passed, on the motion of the Rev. T. W. Turner, seconded by Captain Jessel, M.P.

### BRITISH LYING-IN HOSPITAL.

The annual meeting of the Governors of the British Lying-in Hospital, Endell Street, W.C., was held at the hospital on the 22nd inst. at five p.m. Mr. C. E. Farmer, chairman of the Board of Management, presided. In presenting the report Mr. Farmer remarked that it gave him great pleasure to see a larger number of Governors with them than usual. It was so much more satisfactory to the board when the subscribers took the opportunity of the annual meeting to come and hear how the institution had been managed and the funds applied. Referring first to the financial position of the hospital, he drew attention to the fact that the adverse balance had been reduced from £505 to £163. The diminution of their debt was due to donations, grants from the Prince of Wales's Hospital Fund and others, a legacy from Mrs. Beach, and fees from nurse pupils. The board gratefully acknowledged this help, but the list of subscribers had been thinned by removals, deaths, and non-payment of subscriptions, and it was desirable that other contributors should be found, as subscriptions were the backbone of a hospital's income. They had had to incur an extraordinary amount of expenditure during the last two years, and further outlay was necessary, as their architect had found the drainage defective. The number of in-patients had risen from 269 in 1898 to 331. Four extra beds had been gained by putting one more bed in each ward, and they were always full. Owing to the change in the character of the neighbourhood by replacing cottages with large buildings, the number of out-patients had only increased from 411 in 1898 to 419. It was not probable that these figures would be augmented. It was a matter of congratulation that there had been no deaths amongst the in-patients, and only two amongst the out. These were probably due to the delay in sending for the matron. In one case this was undoubtedly the cause of death. The hospital was being put on to the National Telephone system, which the managers expected to prove of great service. Mr. Cox seconded the adoption of the report, and it was put to the meeting and carried. A consultation was held by the Board of Management after the meeting with the medical authorities as to the arrangements to be made in order to permit of the reconstruction of the drains. It was decided that the work should be undertaken in two portions, thus enabling the authorities to keep open one-half the hospital during the time the other was closed.

### ST. MARY'S CHILDREN'S HOSPITAL.

The second annual court of governors of St. Mary's Children's Hospital, Plaistow, was held on the 22nd inst., the Venerable Archdeacon of Essex, Canon Stevens, presiding. The accounts presented showed the ordinary income of the year to have been £3,352, and the ordinary expenditure £3,185. The extraordinary income (proceeds of sale room and endowment donation) was £495, and the extraordinary expenditure (repairs and repayment to building fund) £304. The committee reported that the year closed with a small balance in hand after meeting the overdraft of £243 due to the bankers and repaying the building fund £120 of the amount borrowed, leaving £160 still owing. £500 had been received from the Prince of Wales's Fund, of which £200 was given for maintenance and £300 for improvements in the mortuary and laundry. Lord Lister had consented to become the first president of the institution, while Lord Claud Hamilton, Lord Raglan, Mr. A. F. Hills, and Captain Matthews had accepted the office of vice-presidents. On the staff, Mr. J. C. S. Peatson succeeded Mr. W. W. Farnfield as senior resident medical officer, and Mr. R. M. Ralph was appointed assistant resident medical officer. At the close of the year Miss L. K. Given-Wilson resigned the secretaryship of the hospital, a post which she had held since the foundation of the institution in 1893.

In moving the adoption of the report and accounts, the Chairman mentioned that the work of the hospital for the year showed the following comparisons: In 1898 the in-patients were 631; in 1899 they were 569; the fall off in numbers was accounted for by the fact that the patients had been kept rather longer—the average residence being 20 days in 1898 and 22·13 in 1899—and also owing to the wards having to be closed twice for disinfection, cases of scarlet fever having occurred. The average number of beds occupied was 34·5, as compared with 35 in 1898. The cost per week had slightly increased, being £1 3s. 7¼d., as against £1 2s. 1d. in 1898. The number of out-patients had greatly increased, being 14,238, as against 10,094 in 1898.

The motion was agreed to, and the following six representatives of the court of governors were elected to the committee of management, viz.: Captain Matthews, Canon Pelly, Dr. Randall, and Messrs. J. Temperley, Page, and David Howard.

The Rev. Given-Wilson called attention to the desirability of taking steps to extend the institution, which was meeting a great want—much greater than any of them had anticipated when the movement was started. They were in a position to extend so far as the possession of land went, but they lacked funds. The work should be undertaken at the earliest opportunity on account of the poverty of the place, the wants of the people and the inability of the hospital in its present state to meet those wants. He expressed the belief that for £7,000 a beautiful hospital could be built as an extension of the present one. They needed offices, an operating theatre, 30 more beds, so as to double their accommodation; and as a means of saving the expense of lodging, accommodation for their nurses. Some discussion ensued as to ways and means, and eventually it was left to the committee to address themselves to the question.

### DENTAL HOSPITAL.

The forty-second annual meeting of the Dental Hospital of London was held on the 15th inst., Mr. John Fairbank presiding. The report stated that the committee had every reason to hope that before next year their new building would be complete and in occupation, and that therefore this would be the last meeting held on the present premises. The usefulness of the institution was steadily increasing, but, owing to the fact that by the compulsory demolition of the property on the site of the new hospital building, operations were prematurely forced on them, occasioning considerable loss of income from rentals, their financial position was not such as was desired. They were glad to announce that their annual subscriptions had still further increased from £1,307 in 1898 to £1,383. The total receipts for the general fund were £2,988, including £100 legacy. The ordinary expenditure was £2,005, and £1,000 was transferred to the building account. The receipts on account of the latter during the year were £871. Dr. Joseph Walker moved the adoption of the report, which was seconded by Mr. Allen Stoneham, the chairman of the committee, who said he was astonished at the amount of work done for so small an amount of money as they expended in that institution. He thought the fact that during such a year as the past their annual subscriptions had increased was evidence that the public had confidence in them. The Chairman thought they had reason to congratulate themselves on the good work done, but he thought the public did not sufficiently appreciate it. The importance of sound teeth to the general health was not generally realised. He had been told that no less than 25 per cent. of the men applying to enter the navy had to be rejected on account of their teeth. Money could be got for libraries and picture galleries, and yet they could not get the £30,000 or £40,000 for building their new hospital to give them light and room for their work. He was glad to find how well advanced the new building was, and, in the hope that it might stimulate others, he had given the secretary £100, with the understanding that if an additional £800 were collected during the year he would give another £100, and so complete the £1,000. This announcement was received with applause, and after the adoption of the report and accounts the meeting separated with the customary votes of thanks.

### ROYAL LONDON OPHTHALMIC.

The first meeting in the new building in the City Road of the Royal London Ophthalmic Hospital—so long known as the Moorfields Eye Hospital—was held on the 21st inst. The Bishop of London presided, the president of the hospital, Lord Avebury, being absent through illness. At the opening of the proceedings the Secretary read a letter from the treasurer, Mr. John Deacon, announcing his intention to increase his subscription from five guineas to ten guineas, and to give a donation of £200. Mr. Sturgis, the chairman of the Committee of Management, formally presented the report and accounts, from which it appeared that the ordinary income for the year was £3,648, and the ordinary expenditure £8,889. The extraordinary income was £1,259, of which £750 was from the Prince of Wales's Fund, and the remainder legacies. The deficiency for the year was therefore £3,982. The past year, the report stated, had been an eventful one in the annals of the hospital, and marked a new epoch in its history. The work which had been carried on for so many years at the old premises in Blomfield Street, Moorfields, had now been transferred to the new building in the City Road, about a mile away. During the year the Princess of Wales and the Duke and Duchess of York had become patrons. The new building was formally opened on June 27th by the Duke of York, accompanied by the Duchess, but it was not till September 4th that the reception of patients was actually begun. In spite of the enormous work involved in the removal to the new building work was carried on in the old hospital up to August 19th for in-patients and up to August 26th for out-patients, thus leaving in the latter department an interval of only one week. In 1899 the total number of in-patients was 1,919. The out-patients numbered 37,832, a great increase over the preceding year, and the total attendances amounted to 93,200. The inquiry officer was obliged during the year to refuse no less than 466 applicants. Among other donations special mention was made of £50 from Mr. John Tweedy, which, with £100 from a reading given by Sir Squire Bancroft, and £151, the proceeds of a ball at the Empress Rooms, Kensington, had been set apart to form the nucleus of a rent fund, the interest of which was to be devoted to meeting the heavy ground rent of £1,210 a year. The old building and site in Blomfield Street were under agreement for sale, and the proceeds of this would in a great measure defray the cost of the new building and its equipment. Regret was expressed at the deaths of three members of the committee: Mr. Charles Gordon, who for nearly fifty years was a member, and for many years chairman; Mr. Edward Hogg, chairman of the House Committee; and the Rev. William Wardle. The committee also announced with regret the resignation, owing to ill-health, of the matron, Miss Robinson. They have been fortunate in securing the services of Miss Richards, lately on the staff of the London Hospital.

In moving the adoption of the report and accounts, the Chairman said that all who were friends of the institution would feel that a very exceptional interest attached to that meeting. The hospital had been in its present building, and in working order for about half a year. It had settled down in a building in all respects worthy and which enabled it to do its work with much greater efficiency than it ever did before. But although the building was very much better adapted for its purpose than the former one, and although it cost nothing, because it was built with the proceeds of the sale of the former site, yet it carried with it the great disadvantage of not being freehold, and of being saddled with a large ground rent, which amounted to no less a sum than £1,210. That constituted a large permanent drain upon the resources of the hospital. The balance-sheet showed an adverse balance of nearly £4,000, and, of course, that was a very serious matter to face. He believed there was no special hospital which had so great a claim on public sympathy and support as that institution had. (Hear, hear.) The ordinary room and the ordinary house were unfitted for the treatment of diseases of the eye, as there were none which required more care and attention, which were so curable if they were taken in good time, or the ravages of which were so great if they were allowed to go on. They would recall the touching and pathetic words Shakespeare had put into the mouth of Prince Arthur, in "King John," when he feared his eyes were to be put out, appealing to Hubert that had he but felt—

> "a grain, a dust, a gnat, a wandering hair,
> Any annoyance in that precious sense,"

and to the "feeling what small things are boisterous there." There was, indeed, nothing which caused so much pain, which so absolutely unfitted a person for the ordinary avocations of life, which was so distressing, or which, if neglected, became so dangerous to the permanent usefulness of anyone, and he was quite sure if they could make the needs of that hospital more widely known to the large philanthropic public in London, the support that was needful would be provided, and the Committee of Management would be able to look forward with courage to the work of the institution in future. (Hear, hear.)

Mr. Sturgis seconded the resolution. They had indeed, he said, passed through a very important year. It was only with great reluctance they had decided to vacate the old premises and take on the responsibility of building their present home. But they were content with the result, and they felt that now they had a hospital which could vie with any other in the world, and which was equipped with the latest appliances science could suggest. They had a strong committee of management, willing to give time to the end in view, and if the public did but realise how every penny entrusted to them was well spent they would have no difficulty about getting the funds required. But that was where their real difficulty lay. Considering the immense amount of work accomplished, it was sad to think their support was so small. Their annual subscriptions were under £1,000, and their donations less than £900. Indeed, he thought it a disgrace that such an institution should get so little help. It was a matter that concerned the whole nation, but the State was willing to leave it to the public, and the public did not respond. Their pressing need now was for a fund to guarantee their ground rent. If they could get £50,000 subscribed for the purpose, and invested, it would leave their regular income free for the actual work of the hospital.

The resolution was then agreed to. Mr. H. Davison and the Rev. J. W. Pratt were re-elected members of the committee of management; and Messrs. Alan Cadell, Stanley Christopherson, William Muir, and Edward Pollock were added to that body. Sir Squire Bancroft, in recognition of his services to the hospital, was elected an honorary life governor. It was also resolved: "That the election of honorary medical and surgical officers be vested in a committee, to consist of the committee of management, together with six members of the medical board, seven members of the election committee to form a quorum, of whom not less than four shall be members of the committee of management; and that any candidate personally soliciting votes shall be deemed to be disqualified. Such committee to hold power until the next annual meeting of the governors." Mr. John Griffiths and Mr. H. Woodburn Kirby were elected auditors; votes of thanks, very cordially proposed, were voted to the medical and surgical officers, the clerical staff—the secretary especially being warmly eulogised—the nurse, and the editors of the metropolitan journals for their aid; and the customary compliment to the chairman brought the meeting to a close. Many of those present availed themselves of the opportunity of inspecting the new building, which was greatly admired.

